# Event and Comment

**Published:** 1919-05

**Authors:** 


### DENTAL REGISTER

THE MAGAZINE OF DENTISTRY

Vol. LXXIII MAY, 1919 No. 5

### EVENT AND COMMENT

**Standardizing Technical Dentistry** For many years there has been a frequently expressed desire, especially by college instructors, that some methods of generally accepted procedure be incorporated into a pedagogic system in order that more harmonious instruction may be given in dental colleges. In order that this might be accomplished most effectively, special organizations have been promoted that dentists who confine their practices largely to one line of effort, might concentrate their studies on a single branch of practice, and so reach some definite conclusions as to the best methods. Orthodontia perhaps serves as one of our best illustrations of what has been accomplished in this way, because it has been most consistently practiced as a specialty, and more determination has been put into the endeavor to establish a definite technical procedure in this department of practice. While this effort at standardization has been fairly successful in orthodontia practice, there are at the present time, at least three more or less distinct methods of instruction and procedure, with so many individual variations as to indicate confusion still more or less prevalent. The recent endeavor to establish a standard method of treating pulpless teeth, is another example of how wide a variation there is in ideas and methods, and how slow is the progress toward a common procedure. Dr. Black, a few years ago, thought he could standardize cavity preparation and instruments used in operative dentistry, but it only requires a casual inspection of our text-book on operative dentistry to see that although much has been accomplished, the entire subject is far from a condition of uniform procedure.

The most recent effort at standardization is the organization of a Society of Prosthodontists with the purpose in view of getting the teachers together for a conference on the best methods of practice. While this organization is not expected to standardize the prosthetic material for instructional purposes especially, its findings will undoubtedly be of great advantage to teachers of this subject. Prosthetic procedures are becoming more important every day and there is not only great need of improving our technic in this department of practice, but the whole subject needs a closer study than has ever been given to it. The professional bearings of this department of practice too generally have been ignored, and little thought has been bestowed on any part of this work other than the mechanical or technical side of it. This subject, like all others, must finally rest upon a basis of actual facts that can be demonstrated with reasonable or convincing acuracy. It is so largely a technical art that it is impracticable to unify the art features, just as it is impossible to erect a formula for the sculptor or painter. There are, however, some fundamentals about which a reasonable degree of dogmatism is practicable, and it is to be hoped that in this new organization there shall be no tolerance of endeavors to exploit favorite methods of technical procedures that can not rest on the best scientific information we possess, or upon which our scientific knowledge may not be modified, or more securely established. There is a grand opportunity for this society to do a work which will demonstrate to our other technical departments the possibility of having more accurate data as to the fundamental principles of our technical art than we seemingly think possible. If we could standardize technical teaching on the basis of a few of the fundamentals scientifically determined, we should have a basis from which to work that would serve to create a new interest in the subject, and also predetermine to a large extent the character of our ideals and practice in the future. Let us hope therefore there may be no waste of time or effort in fruitless discussions of means and methods that are purely personal and of improbable scientific consequences.

**Dental Hygienist Legislation** Another state, Michigan, has enacted a law legalizing the Dental Hygienist. The legislation is a part of the new dental law which was passed without opposition and at the almost unanimous request of the dental profession. The growth of the school work has forced this issue upon the profession, as there is at this time no other probable solution for handling this matter. Owing to the shortage of dentists of the present time, and the growing demand of the people for this form of preventive treatment, it seems the only practicable way in which this work can be adequately accomplished. This means that Michigan will have to provide a training school for the dental hygienist, as there is no other way in which she can be provided. Let us hope this matter may be put on an educational basis that is comparable with the preparation now required for the professional dentist.

